# Metastatic lymph node characteristics as predictors of recurrence/persistence in the neck and distant metastases in differentiated thyroid cancer

**DOI:** 10.1590/2359-3997000000307

**Published:** 2017-12-01

**Authors:** Mayara Peres Barbosa, Denise Momesso, Daniel Alves Bulzico, Terence Farias, Fernando Dias, Roberto Araújo Lima, Rossana Corbo, Mario Vaisman, Fernanda Vaisman

**Affiliations:** 1 Universidade Federal do Rio de Janeiro Departamento de Endocrinologia Rio de Janeiro RJ Brasil Departamento de Endocrinologia, Universidade Federal do Rio de Janeiro (UFRJ), Rio de Janeiro, RJ, Brasil; 2 Instituto Nacional de Câncer Departamento de Endocrinologia Rio de Janeiro RJ Brasil Departamento de Endocrinologia, Instituto Nacional de Câncer (Inca), Rio de Janeiro, RJ, Brasil; 3 Instituto Nacional de Câncer Serviço de Cirurgia de Cabeça e Pescoço Rio de Janeiro RJ Brasil Serviço de Cirurgia de Cabeça e Pescoço, Instituto Nacional de Câncer (Inca), Rio de Janeiro, RJ, Brasil; 4 Instituto Nacional de Câncer Rio de Janeiro RJ Brasil Instituto Nacional de Câncer (Inca), Rio de Janeiro, RJ, Brasil; 5 Instituto Nacional de Câncer Universidade Federal do Rio de Janeiro (UFRJ) Departamento de Endocrinologia Rio de Janeiro RJ Brasil Departamento de Endocrinologia, Instituto Nacional de Câncer (Inca), Rio de Janeiro, RJ, Brasil e Departamento de Medicina Nuclear, Universidade Federal do Rio de Janeiro (UFRJ), Rio de Janeiro, RJ, Brasil; Universidade Federal do Rio de Janeiro (UFRJ) Departamento de Medicina Nuclear Rio de Janeiro RJ Brasil

**Keywords:** Neck recurrence/persistence, thyroid cancer, lymph nodes, prognosis

## Abstract

**Objective:**

The aim of this study was to evaluate the association between this characteristic and outcomes in patients with lymph node metastasis in a Brazilian cohort.

**Subjects and methods:**

This study examined a retrospective cohort of adult patients diagnosed with differentiated thyroid cancer and lymph node metastases from 1998 to 2015 in two referral centers. Number, location, size and extranodal extension (ENE) of metastatic lymph nodes were assessed and correlated with response to initial therapy.

**Results:**

A greater number of metastatic nodes, larger size, presence of lateral neck disease and ENE were all associated with a lower probability of achieving an excellent response to initial therapy (p ≤ 0.05 for all these parameters). Local recurrent disease had a significant association with lymph node number (6 in the recurrence/persistence group versus 4 in the non-recurrent group; p = 0.02) and ENE (19.2 versus 75%, p = 0.03). Lateral neck disease was the only characteristic associated with distant metastasis and was present in 52.1% of the group without metastasis and 70.4% of the group with metastasis (p = 0.001).

**Conclusion:**

The lymph node characteristics were associated with response to initial therapy and neck recurrence/persistence, confirming the importance of the analysis of these factors in risk stratification in a Brazilian population and its possible use to tailor initial staging and long term follow-up.

## INTRODUCTION

Differentiated thyroid cancer (DTC) is the cancer with the highest increase in the incidence in the United States (1). Cervical lymph nodes are the most common site of metastases. In most series, the incidence varies from 20-50%, depending on tumor size, age, gender and local invasion; however, it can be found in up to 90% in countries that routinely adopt prophylactic neck dissection (2).

The prognostic significance of lymph node metastases in DTC is still controversial (3). Most studies show that the presence of lymph node metastases has little impact on overall survival, being more significant in older patients despite a great impact on recurrence/ persistence rates and impairment of quality of life in all age groups (4,5).

In the past, the presence or absence of node metastasis and its location in the neck were the only factors analyzed to classify node disease (6). Recently, lymph node characteristics such as number, size, location and extranodal extension (ENE) have been shown to have great impacts on the risk of nodal disease recurrence/ persistence (7). In 2015, the American Thyroid Association (ATA) recognized the importance of these factors and recommended that patients be considered as low risk when there is no evidence of clinical nodal metastases (cN0) or when micrometastases (less than two millimeters) in five or fewer lymph nodes is present. Patients with clinically evident lymph nodes (cN1) and/or more than five lymph nodes, all less than three centimeters, should be classified as intermediate risk. The Committee defined high risk patients as those with lymph nodes larger than three centimeters. The presence of ENE was not included as an independent factor, but the presence of more than three lymph nodes with ENE was considered a high risk feature with a 40% risk of recurrence/persistence (2). The clinical implication of this new stratification has also an impact in adjuvant radioiodine (RAI) therapy, allowing low nodal volume disease to be managed without adjuvant RAI therapy, for example (3). However, data in the literature validating this impact around the world is still scattered.

The aim of this study is to evaluate the association between the characteristics of metastatic lymph nodes and the final clinical status according to response to therapy. In addition, this study aims to analyze the association of these characteristics with cervical recurrence/persistence and distant metastatic disease risks.

## SUBJECTS AND METHODS

The study is a retrospective analysis of a cohort of patients 21 years of age or older diagnosed with DTC with lymph node metastases from 1998 to 2015. Data were obtained from the University Hospital Clementino Fraga Filho (UFRJ) and the Brazilian National Cancer Institute (Inca). Patients were followed for at least 1 year, and all were submitted to total thyroidectomy and RAI therapy. The RAI activity was decided by a multidisciplinary team based on clinical, histopathological and complementary tests. In neither institution is it routine to perform prophylactic cervical dissection. Patients with a diagnosis of medullary, anaplastic carcinoma and poorly differentiated variants such as insular, tall and columnar cells were excluded.

### Laboratory studies

Between 1998 and 2001, a thyroglobulin (Tg) assay with a functional sensitivity of 0.5 ng/mL was employed. From 2001 until 2010, serum Tg was quantified by an immunometric assay (Immulite) with a functional sensitivity of 0.2 ng/mL. From 2010 until the present, the functional sensitivity was reduced to 0.1 ng/mL.

### Evaluation of outcomes

Clinical-pathological characteristics of the patients, treatment details (surgery, RAI therapy) and postoperative follow-up (Tg, recurrence/persistence, deaths) were obtained. The characteristics of the metastatic lymph nodes were analyzed such as number, location, size of the largest lymph node and presence of ENE. Patients were classified by AJCC/TNM (8) and ATA risk classification (2). Response to initial therapy was assessed by ATA and classified as follows: excellent response (negative imaging and suppressed Tg < 0.2 ng/mL and stimulated Tg < 1.0 ng/mL); indeterminate response (nonspecific findings on imaging studies, nonstimulated Tg detectable but < 1 ng/mL, stimulated Tg detectable but < 10 ng/mL); biochemical incomplete response (negative imaging and nonstimulated Tg > 1 ng/mL or stimulated Tg > 10 ng/mL); or structural incomplete response to therapy (structural or functional evidence of disease, with any Tg level) (2).

Patients were classified in the final follow-up as having no evidence of disease when the suppressed Tg was less than 1 ng/mL, no antibodies were present, and there was no structural evidence of the disease. Patients with suppressed Tg greater than 1 ng/mL and stimulated greater than 2 ng/mL or any evidence of structural disease (complementary exams or biopsy) were classified as biochemical or structural persistence, respectively. Cervical recurrence/persistence was defined as follows: positive cytology/histology, highly suspicious lymph nodes or thyroid bed nodules on the US (hyper-vascularity, cystic areas, heterogeneous content, rounded shape and enlargement on followup), or cross-sectional imaging highly suspicious for metastatic disease. Distant metastases were assessed by cross sectional images and considered as present when there was iodine uptake and/or were highly suspicious on CTs and/or MRIs, even with no iodine uptake but with high thyroglobulin levels or if proven by biopsy.

The ethical boards of both institution involved approved this study.

### Statistical analysis

Continuous data are presented as the mean and standard deviations with median values. For comparing nonparametric medians, the Mann-Whitney test was used, and for categories, we used Chi-square and Fisher's exact tests. Analysis was performed using SPSS software (Version 20.0 for MAC; SPSS, Inc., Chicago IL).

## RESULTS

General characteristics of the participants are shown in Table 1. As expected, the majority were women (71.1%), and the median age was 41 years. Two hundred and eight patients (98,57%) had a diagnosis of papillary carcinoma, of which 90.9% (192) were the classical form, 6.2% (9) were follicular variant and 1.4% (3) were Hürthle cell variant. Only 3 patients (1,4%) were diagnosed as having follicular carcinoma. The median size of the tumors was 2.2 cm (0,3-15), 121 patients (57.3%) had tumors with extrathyroid extension, 103 (48.8%) had multifocality and 63 (29.9%) had vascular invasion. Regarding the characteristics of the lymph nodes, 131 patients (62.1%) had N1 tumors, 99 (46.9%) had more than five metastatic lymph nodes, 40 (19%) had the largest lymph node affect above or equal to 3 cm, and 15 (7.1%) patients had descriptions of extranodal extension (ENE). Ninety one patients (43.1%) had only involvement of the central compartment (N1a), and 102 (48.3%) had lateral and central compartment involvement (N1b). Twenty- seven patients (12.8%) presented distant metastases in diagnosis. All patients underwent RAI treatment. The stratification of patients according to ATA 2015 risk showed 21 patients (9.9%) were low, 110 (52.1%) intermediate and 80 (38%) high risk.

**Table 1:** Cohort description

Characteristic		N = 211	%
Gender (F:M)		150:61	71.1:28.9
Age at diagnosis (years)		41 (21-77)	-
Size (cm)		2.2 (0.3-15)	-
Race			
	White	146	69.2
	Black	10	4.7
	Latin	51	24.2
	Not informed	4	1.9
Size (cm)		2.2 (0.3-15)	-
Total thyroidectomy		211	100
Histology			
	PTC	192	90.9
	Invasive follicular variant of PTC	13	6.2
	Hurthle variant	3	1.4
	FTC	3	1.4
ETE		121	57.3
Multifocality		103	48.8
Vascular invasion		63	29.9
cN1		131	62.1
Lymph node staging			
	> 5 metastatic lymph nodes	99	46.9
	> 3 cm metastatic lymph node	40	19
	Central compartment only (N1A)	91	43.1
	Lateral compartment (N1B)	102	48.3
	Extra nodal extension	15	7.1
Distant metastasis		27	12.8
RAI		211	100
Initial activity (mCi)		150 (30-250)	-
Total activity (mCi)		200 (100-1100)	-
ATA 2015 risk			
	Low	21	9.9
	Intermediate	110	52.1
	High	80	38
Follow-up (years)		6.2 (0.8-17)	-
Final status			
	NED with additional therapy	89	42.2
	Biochemical persistent disease	74	35.1
	Structural persistent disease	43	20.4
Deaths		5	2.4

PTC: papillary thyroid cancer; FTC: follicular thyroid cancer; ETE: extrathyroidal extension; NED: no evidence of disease; ATA: American Thyroid Association; RAI: radioactive iodine.

As also shown in Table 1, the median follow-up was 6.2 years (0.8-17). At the end of follow-up, 89 patients (42.2%) were disease-free, 74 (35.1%) maintained biochemical disease, 43 (20.4%) had structural persistence, and 5 (2.4%) died from the disease.

Table 2 shows the relationship between lymph node characteristics and response to initial therapy. The number of lymph nodes, size, presence of ENE and N1b involvement presented greater association with an incomplete structural response, whereas the presence of metastatic node exclusively in the central compartment (N1a) was associated with a higher chance of having an excellent response.

**Table 2:** Lymph node characteristics versus response to initial therapy

Characteristic	Excellent response (n = 67)	Indeterminate response (n = 16)	Biochemical incomplete (n = 84)	Structural incomplete (n = 44)	p-value
> 5 metastatic lymph nodes	33.3%	37.5%	71.5%	70%	0.05
> 3 cm metastatic lymph nodes	16.7%	25%	26.6%	26.7%	0.01
Lymph node	49.3%	50%	47.6%	20.5%	0.02
Central compartment alone (N1A)					
Lateral compartment (N1B)	48.7%	50%	52.4%	79.5%	0.02
Extranodal extension	6.7%	6.25%	8.2%	14.3%	0.03

Patients with recurrent lymph node disease tend to be younger and have a greater number of metastatic lymph nodes, which tend to be more than 3 cm, than patients who did not have neck recurrence/persistence/ persistence of nodal disease. The involvement of lateral neck (N1b) and ENE were also more frequent in patients who recurred. The number and presence of ENE were the features that had significant associations with recurrence/persistence with a relative risk RR 1.3 (IC: 1-2.7) (Table 3).

**Table 3:** Risk factors for lymph node structural recurrence/persistence during follow-up

N = 211		LN Recurrence/persistence (n = 52)	No LN recurrence/persistence (n = 159)	p-value
Age		**38 (21-77)**	**42 (21-72)**	**0.08**
Gender (F:M)		67.3%:32.7%	71.7%:28.3%	0.3 (NS)
cN1		66.7%	75.3%	0.4 (NS)
Number of metastatic lymph nodes		**6 (2-33)**	**4 (1-30)**	**0.02**
> 3 cm metastatic lymph node		**30.5%**	**26%**	**0.08**
Lymph node		**28.5%**	**41.6%**	**0.07**
Central compartment (N1A)				
Lateral compartment (N1B)		**71.5%**	**58.4%**	**0.07**
Extranodal extension		**19.2%**	**7.5%**	**0.03**
Histology				0.4
PTC		90.6%	86.4%	
	FV PTC	7.5%	9.3%	
	Hurthle variant	0	2.9%	
	FTC	1.9%	1.4%	
Tumor size (cm)		2.5 (1.0-8.0)	2.0 (0.3-15)	0.3 (NS)
ETE		65.3%	55.8%	0.3 (NS)
Multifocality		53.1%	51.1%	0.8 (NS)
Vascular invasion		**34%**	**20.4%**	**0.05**
Distant metastases		13.5%	12.6%	0.5 (NS)
RAI initial activity (mCi)		150 (100-200)	150 (30-250)	0.5 (NS)
RAI cumulative activity(mCi)		**350 (120-1050)**	**150 (100-1100)**	**< 0.001**
Post-operative suppressed Thyroglobulin (ng/mL)		**92 (< 0.1-460)**	**1.15 (< 0.1-280)**	**0.01**

PTC: papillary thyroid cancer; FTC: follicular thyroid cancer; ETE: extrathyroidal extension; NED: no evidence of disease; ATA: American Thyroid Association; RAI: radioactive iodine.

Regarding the primary tumor, vascular invasion was the only feature that was associated with lymph node recurrence/persistence. As expected, the recurrent group had a higher basal Tg and underwent a higher cumulative activity of RAI overtime.

Finally, as shown in the Table 4, N1b tumors and the presence of vascular invasion presented a statistically significant association with the presence of distant metastases. Other lymph node characteristics were not significant as risk factors for distant metastases.

**Table 4:** Risk factors for distant metastasis

	Distant metastasis (n = 27)	No distant metastasis (n = 184)	RR (IC 95%)	p-value
Clinical N1	87.5%	71.4%	1.19 (0.99-1.5)	0.06
Vascular invasion by the primary tumor	50%	28.2%	**1.83 (1.19-2.82)**	**0.04**
> 3 cm metastatic lymph nodes	22%	19.5%	1.13 (0.52-2.4)	0.7
Extra nodal extension	12%	9.1%	1.2 (0.39-4.07)	0.7
> 5 metastatic lymph nodes	59%	44.5%	1.3 (0.93-1.8)	0.1
N1b	70.4%	52.1%	**1.3 (1.01-1.78)**	**0.03**

## DISCUSSION

The present study analyzed the characteristics of tumor and metastatic lymph nodes in patients with differentiated thyroid cancer (DTC), associating them with response to initial therapy, lymph node recurrence/ persistence and the presence of distant metastases. In this study, the number of metastatic nodes (> 5) and the presence of ENE were significantly associated with neck recurrence/persistence. In addition, their size and location showed a trend to be associated with an increased risk of neck recurrence/persistence during follow-up. Hence, the presence of lateral neck disease was also associated with a greater risk of distant metastases.

Our first analysis aimed to correlate the metastatic lymph node features at diagnosis with the response to initial therapy within the first 6-24 months of follow-up. All lymph node characteristics studied, except N1a, had a high correlation with incomplete structural or biochemical responses. Other studies in the literature, which also analyzed response to therapy and lymph node characteristics, showed similar results. Jeon and cols. (10) classified their patients according to the number and size of lymph nodes (very low risk when there are 5 or less and they are less than 0.2 cm, low risk when there are 5 or less and they are 0.2 cm or greater, and high risk when there are more than 5) and concluded that these features predicted the response to initial therapy. In the study by Lango and cols. (11), the presence of ENE reduced the likelihood of an excellent response (OR 3.5 CI 95% 1.3-10; p 0.01), and increased the likelihood (OR 5 CI 1.2-21; p 0.03) of a Tg postoperative greater than 50 ng/mL.

The present study also demonstrated a significant correlation between the number of metastatic lymph node and recurrent disease (p = 0.02). Similarly, Lee and cols. demonstrated that the higher the number of metastatic lymph nodes the higher the recurrence/ persistence rate: patients with 2 to 5 metastatic lymph nodes showed 2 to 3 times greater recurrence/ persistence than patients with one, and patients with 6 or more metastatic lymph nodes had a risk of 3.7 times greater (12). Ricarte-Filho and cols. demonstrated that the number of metastatic lymph nodes (greater than 3 in patients aged 45 years or less and greater than 5 in those over 45 years) was an important and independent, predictor of recurrence/persistence-free survival (13). Sugitani and cols. and Ito and cols. showed that the presence of 5 or more metastatic lymph nodes was an independent predictor of recurrence/persistence but only in young patients (9,14). Randolph and cols. published a meta-analysis in which the presence of less than 5 lymph nodes was associated with a median recurrence/persistence rate of 4% (3-8%), versus 19% (7-21%) in patients with more than 5 lymph nodes.

The ATA 2015 guidelines did not include ENE, but the presence of more than 3 lymph nodes with ENE has been considered a high-risk characteristic with a 40% recurrence/persistence rate (1). Previous studies have not shown an association between ENE and outcomes probably due to small samples (15). However, subsequent studies have shown the importance of this characteristic in relation to recurrence/persistence (7,9,11,16,17), as our findings have demonstrated. Lango and cols. showed that patients with ENE had higher post-treatment Tg levels as well as a higher risk of persistent nodal disease and progression of systemic disease; the presence of ENE was associated with a 20% risk of persistent nodal disease (11). Leboulleux and cols. showed that more than 3 metastatic lymph nodes with ENE were associated with a high recurrence/ persistence rate in patients with DTC when compared to patients with less than 3 (18). However, the study by Ito and cols. did not demonstrate an increased risk of recurrence/persistence in patients with ENE, although the number of lymph nodes with this characteristic was not evaluated (19). In our study, 19.2% of the patients with lymph node recurrence/persistence had ENE versus 7.5% in the patients without recurrence/ persistence (p = 0.03). One of the major limitations in the ENE analysis is the interobserver variation of its definition. Du and cols. evaluated the concordance rate among 11 pathologists from the United States, Canada and Italy and the concordance ratio was 0.68. This low rate may explain the variation of studies in the analysis of the prognosis of ENE (20).

Furthermore, this study also analyzed the correlation between distant metastases and metastatic lymph node characteristics. In our Brazilian cohort, the presence of lateral neck lymph node metastases at diagnosis was the most important factor associated with a greater risk of having distant metastases, as assigned by TNM (8). Similarly, Chen and cols. (21) found that the presence of a lateral metastatic lymph node was an independent risk factor for distant metastasis (OR 4.02, 95% CI 1.2512.95; p: 0.02). Lango and cols. (10) demonstrated that the impact of ENE on the risk of developing distant metastases was independent of nodal persistence (HR 4.3 CI 95%1.2-15; p: 0.02), and Yamashita and cols. (22) showed that the presence of ENE is associated with the presence of distant metastases (p < 0.0001). The absence of association between ENE and distant metastasis in our study may be due to the small number of patients in whom this characteristic was analyzed.

In conclusion, this study showed association between lymph node characteristics and outcomes such as recurrence/persistence and response to initial therapy, reinforcing the importance of the analysis of these factors for stratification and therapeutic management.
